# Effect of Chitosan Nanoparticles as an Irrigant in Regenerative Endodontic Therapy of Necrotic Immature Permanent Teeth: An In Vivo Dog Model Study

**DOI:** 10.3390/biomedicines14051041

**Published:** 2026-05-03

**Authors:** Safwat Elwaseef, Huda Ibrahim Mostafa, Abeer Ezat Wahba, Ahmed Mostafa Abbas, Ashraf Mohamad Emran, Gladistone Cadete Meros, Tarsyo Marcel Silva Montezuma, Ehab Hamed Mostafa Elwardaney

**Affiliations:** 1 Department of Endodontics, Faculty of Dental Medicine, Al-Azhar University, Asuit 71524, Egypt; 2 Advanced Education in General Dentistry, Eastman Institute for Oral Health, University of Rochester, Rochester, NY 14620, USA; 3 Department of Endodontics, Faculty of Dental Medicine for Girls, Al-Azhar University, Cairo 11754, Egypt; drhudaabdo2010@azhar.edu.eg; 4 Department of Oral and Dental Biology, Faculty of Dental Medicine for Girls, Al-Azhar University, Cairo 11884, Egypt; abeermoghazy.26@azhar.edu.eg; 5 Department of Oral Biology, Faculty of Dental Medicine, Al-Azhar University, Cairo–Boys, Cairo 11884, Egypt; dentist.ahmedmostafa@gmail.com (A.M.A.); ashrafemran88@gmail.com (A.M.E.); 6 Department of Oral Biology, Faculty of Dental Medicine, Al-Azhar University, Asuit–Boys, Asuit 71524, Egypt

**Keywords:** regenerative endodontic, pulp revascularization, chitosan *NPs*, nanobiomaterials, immature permanent teeth, dog model, histology

## Abstract

**Background:** The aim of this study was to investigate the effect of 2% chitosan nanoparticles (NPs) as an irrigating solution during pulp revascularization of immature dog teeth using histological and histomorphometric analyses. **Materials and Methods**: Pulp necrosis and periapical pathosis were induced in 52 incompletely formed roots in four dogs (6–8 months age). These teeth were randomly allocated to Group I (n = 20; irrigation with NaOCl + EDTA) and Group II (n = 20; irrigation with NaOCl + chitosan *NPs*); DAP was used as a medication in both groups. Positive control (6 roots): teeth with induced periapical infections, no treatment procedure, and left open. Negative control (6 roots): teeth that were left untreated for the normal maturation process. Each experimental group was subdivided into two subdivisions in accordance with the post-treatment evaluation periods (1–3 months). The experimental teeth were re-entered following the infection period and disinfected using the assigned irrigation and medication protocol, and the access cavities were sealed. After the evaluation period, medication was removed, and blood clot formation was created through over-instrumentation. Mineral trioxide aggregate (MTA) was applied, followed by glass ionomer restoration (GIC). **Results**: At both 1 and 3 months, Group II demonstrated significantly superior histological organization and higher collagen-positive area percentages compared with Group I (*p* < 0.01), while the negative control showed the highest values and the positive control the lowest. **Conclusions:** Irrigation with 2% chitosan *NPs* significantly improved regenerative outcomes compared with the conventional NaOCl/EDTA protocol in immature canine teeth.

## 1. Introduction

The dental pulp is a specialized connective tissue confined within rigid walls, where it maintains tooth vitality through sensory, immune, and reparative functions. In immature permanent teeth, maintaining pulp vitality is critical for continued root development and apical closure. When pulp necrosis occurs due to trauma or infection, root maturation is arrested, resulting in open apices and thin dentinal walls that increase fracture susceptibility. Conventional apexification using calcium hydroxide or MTA can induce apical barrier, but does not allow for further root maturation or re-establish the normal functions of the dentin–pulp complex (DPC). Consequently, treated teeth often remain structurally compromised [1,2,3,4,5].

Regenerative endodontic therapy (RET) has been introduced as a biologically based alternative aiming to restore pulp vitality and support continued root formation. In this context, re-establishing blood supply within a disinfected canal, often termed pulp revascularization, is a key early event. However, vascular ingrowth alone does not guarantee full biological regeneration. Functional restoration of the DPC requires organized alignment of odontoblast-like cells along the dentinal walls, reinnervation, and repopulation by stromal and progenitor cells capable of maintaining long-term tissue homeostasis. True regeneration therefore entails the reconstitution of structurally organized and functionally competent pulp tissue [2,3,4,5,6,7].

A central challenge in RET is achieving effective canal disinfection while preserving the viability of stem cells and the integrity of dentin-derived signaling molecules. Thorough three-dimensional disruption of microbial biofilms and endotoxins is essential to permit stem cell survival and tissue ingrowth. Sodium hypochlorite (NaOCl) is the most commonly used irrigant because of its strong antimicrobial and tissue-dissolving properties, yet it can be cytotoxic to stem cells of the apical papilla (SCAPs), especially at higher concentrations. Ethylenediaminetetraacetic acid (EDTA) is frequently used to remove the smear layer and liberate dentin-derived growth factors but may cause excessive demineralization and has been associated with reduced stem cell viability under certain conditions. Thus, a major obstacle in RET is to control infection adequately while maintaining the cellular and extracellular matrix components required for regeneration [8,9,10].

To address this challenge, increasing attention has been directed toward bioactive materials that combine antimicrobial efficacy with favorable biological interactions. Chitosan, a naturally derived cationic polysaccharide obtained from chitin deacetylation, has attracted considerable interest in dental biomaterials research due to its biocompatibility, biodegradability, low toxicity, and broad-spectrum antimicrobial activity. In addition, chitosan exhibits anti-inflammatory properties and has been shown to enhance cell adhesion, proliferation, and extracellular matrix formation. These biological characteristics have led to its application in various fields of tissue engineering, including bone, cartilage, and dental tissue regeneration [11,12,13].

Recent advances in nanotechnology have further expanded the therapeutic potential of biomaterials used in regenerative dentistry. Materials engineered at the nanoscale (1–100 nm) possess unique physicochemical properties, including increased surface-to-volume ratio and enhanced surface reactivity, which improve biological interactions with cells and tissues. *NPs* may facilitate deeper penetration into dentinal tubules, targeted antimicrobial activity, and controlled release of bioactive molecules. Incorporating chitosan into NP formulations may therefore enhance its antimicrobial properties while simultaneously improving its regenerative potential through better cell–material interactions and signaling pathways [14,15,16].

Despite the growing interest in chitosan-based biomaterials, most previous investigations have primarily focused on its bulk or hydrogel formulations, with limited attention directed toward the distinct biological advantages conferred by nanoscale systems in regenerative endodontics. Furthermore, the potential application of chitosan NPs as an irrigating agent during RET remains largely unexplored [16,17,18]. Therefore, the present study aimed to evaluate the effect of chitosan NPs used as an irrigant during pulp revascularization in immature canine teeth through histological and histomorphometric analysis. The novelty of this investigation lies in assessing whether nanoscale chitosan incorporated into a regenerative irrigation protocol can influence the structural organization and characteristics of newly formed tissues within the root canal system. The null hypothesis tested was that the use of chitosan NPs during RET would not result in statistically significant differences in histological or histomorphometric outcomes compared with the conventional irrigation protocol.

## 2. Materials and Methods

### 2.1. Animal Housing

This study was conducted on fifty-two immature roots obtained from four clinically healthy dogs aged 6–8 months and weighing between 10 and 15 kg, with equal representation of both sexes. Prior to the experimental procedures, the animals underwent a two-week acclimatization period. During this time, each dog was carefully examined by a qualified veterinarian to ensure overall health and absence of systemic or oral disease. The dogs were housed individually in properly ventilated cages at the Department of Veterinary Surgery, Veterinary Hospital, El Abbasia. Environmental conditions, including humidity, were carefully controlled, and the animals were maintained on a soft diet throughout the study period. All efforts were taken to reduce animal use and minimize discomfort in accordance with accepted welfare standards. The experimental protocol complied with institutional ethical guidelines and received approval from the Research Ethics Committee (REC) of the Faculty of Dental Medicine, Al-Azhar University, Assiut (Approval Code: AUAREC2025001-4).

### 2.2. Sample Size Calculation

Sample size estimation was performed using G*Power software (version 3.1.9.2; Heinrich Heine University, Düsseldorf, Germany) [19]. The calculation was based on a large effect size (f = 0.48) derived from previously published data [20]. With a significance level (α) of 0.05 and a Type II error probability (β) of 0.20 (power = 80%), the minimum required total sample size was calculated to be 52 specimens. Accordingly, the samples were allocated as follows: 40 roots were assigned to the experimental groups (n = 20 per group), and 12 roots were assigned to the control groups, equally divided into positive and negative controls (n = 6 each).

### 2.3. Sample Selection and Grouping

A total of 52 immature permanent roots were randomly assigned to two experimental groups and one control group.

- Group I (n = 20) was irrigated with 20 mL of 1.5% NaOCl for 5 min followed by 20 mL of 17% EDTA for 5 min. The 1.5% NaOCl solution was prepared by diluting 5.25% NaOCl with distilled water at a ratio of 1:3.5 (*v*/*v*). No mechanical instrumentation was performed. Then final irrigation was completed using 5 mL of sterile saline solution.
- Group II (n = 20) received irrigation with 20 mL of 1.5% NaOCl for 5 min followed by 20 mL of 2% chitosan *NPs* for 5 min.

In both experimental groups, a double antibiotic paste (DAP) was used as an intracanal medicament. The paste was prepared by crushing one 250 mg tablet of ciprofloxacin and one 500 mg tablet of metronidazole separately, then mixing the powders in a 1:1 ratio. Distilled water was added as a vehicle in a proportion of one part liquid to five parts powder to obtain a creamy consistency.

- The control group (n = 12) was subdivided into a positive control subgroup (n = 6), consisting of teeth with induced periapical infection left open and untreated, and a negative control subgroup (n = 6), consisting of untreated healthy teeth allowed to undergo normal root development.

Each experimental group was further divided according to the evaluation period at 1- and 3-months post-treatment. Although multiple samples were obtained from the same animal, all teeth were randomly assigned to experimental groups and treated as independent observations, consistent with commonly used experimental designs in similar preclinical endodontic studies.

### 2.4. Preparation of 2% Chitosan NPs

Chitosan NPs were prepared using the ionic gelation method with sodium tripolyphosphate (TPP) as a cross-linking agent. This technique is based on electrostatic interaction between the positively charged amino groups of chitosan and the negatively charged phosphate groups of TPP, resulting in NP formation while minimizing residual acetic acid through ionic cross-linking. Chitosan NPs produced via ionic gelation possess nanoscale dimensions (1–100 nm) and a positive surface charge derived from protonated amino groups, while their high surface area-to-volume ratio and charge characteristics enhance their interaction with microbial cell walls and biological tissues, contributing to their antimicrobial and bioactive performance [16,17].

A 2% chitosan NP concentration was selected based on previous evidence demonstrating a favorable balance between antimicrobial activity, biocompatibility, and stem cell viability. Chitosan NP prepared by this method are typically characterized by nanoscale size and a positive surface charge, facilitating interaction with negatively charged bacterial cell walls. To prepare the solution, 2 g of chitosan powder was dissolved in 1% (*v*/*v*) acetic acid under continuous magnetic stirring at room temperature until a clear homogeneous solution was obtained. A 0.8% TPP solution was prepared separately by dissolving 80 mg of TPP in 10 mL of deionized water. The TPP solution was then added dropwise to the chitosan solution under magnetic stirring at a volume ratio of 2.5:1 (chitosan: TPP). The mixture was further homogenized using a Polytron homogenizer at 5000 rpm to promote uniform nanoparticle formation. Following stirring, three phases were observed: visible aggregates, a clear solution, and an intermediate opalescent suspension. The opalescent zone indicated successful nanoparticle formation due to light scattering by nanosized particles. The suspension was centrifuged at 13,000 rpm for 4 min to isolate the *NPs*. The supernatant was discarded, and the collected *NPs* were thoroughly washed with distilled water to remove residual reagents [21,22,23,24,25].

### 2.5. Procedures

All endodontic interventions were carried out by two well trained endodontists following standardized aseptic protocols to reduce operator-related bias. Prior to treatment, radiographic examination was used to verify incomplete root development in all teeth included in the experimental groups.

#### 2.5.1. First Treatment Session (Induction of Periapical Pathosis)

All procedures were carried out using sterile instruments under strict aseptic conditions. The dogs were fasted for 12 h prior to anesthesia. Premedication was administered 15 min before anesthesia using atropine sulfate (0.05–0.1 mg/kg, subcutaneously) and xylazine HCl (1 mg/kg, intramuscularly). General anesthesia was induced with ketamine HCl (5 mg/kg) administered intravenously through the cephalic vein using a cannula. Anesthesia was maintained with 2.5% thiopental sodium administered intravenously (2.5–25 mg/kg, dose to effect) with additional supplemental boluses (3 mg/kg) when required to maintain adequate anesthesia [26].

After adequate anesthesia was achieved, endodontic access cavities were prepared in both the experimental and positive control teeth. The pulp chambers were exposed using size 2 diamond burs in low-speed handpieces. In each quadrant of every dog, conventional access cavities were prepared in the premolar teeth. The pulp tissue inside the root canals was mechanically disrupted using a sterile size #40 file. Dental plaque collected from the dogs was mixed with sterile saline solution to prepare a suspension. Cotton pellets soaked in this suspension were placed into the pulp chambers of all prepared teeth, and the access cavities were left open for two weeks to allow the development of periapical pathosis. Postoperatively, paracetamol (100 mL containing 1000 mg) was administered once daily as an analgesic, and the animals were monitored daily for signs of pain [27,28].

#### 2.5.2. Second Treatment Session (Disinfection)

After the two-week infection period, the previously infected experimental teeth were re-entered under strict aseptic conditions. The oral cavity was disinfected using Betadine swabbing, cotton roll isolation, and premedication with subcutaneous atropine sulfate injections to reduce salivation. The root canals were disinfected according to the assigned irrigation protocol for each experimental group. Following irrigation and DAP medication, the access cavities were sealed with GIC, and the teeth remained sealed for an additional two weeks [29].

#### 2.5.3. Third Treatment Session (Blood Clot Formation)

After the two-week evaluation period, the teeth in Groups I and II were re-entered under general anesthesia. The canals were first irrigated with 10 mL of sterile saline to remove the DAP. Bleeding into the canal system was then induced by over-instrumentation using a sterile #40 K-file, which was intentionally extended beyond the apical foramen into the periapical tissues to stimulate blood clot formation within the canal. Once a stable blood clot was achieved, MTA was placed over the clot to a thickness of approximately 3–4 mm using an appropriately sized amalgam carrier. Finally, the access cavity was permanently restored with GIC [30].

#### 2.5.4. The Fourth Treatment Session (Euthanasia)

At the predetermined evaluation intervals of one and three months, the animals were humanely euthanized to allow for subsequent histopathological examination. Euthanasia was induced by administering an intravenous overdose of anesthetic (5% ketamine hydrochloride, 20 mL) to achieve deep anesthesia. After confirming profound unconsciousness, a lethal dose of pentobarbital sodium combined with phenytoin sodium was administered to ensure complete euthanasia. Following confirmation of death, 20% buffered formalin (40 mL) was injected via intracardiac perfusion to facilitate tissue fixation. The jaws were then carefully dissected and harvested for subsequent histopathological processing and light microscopic evaluation [26,27,28,29,30].

### 2.6. Histological Evaluation

Following euthanasia, the maxillary and mandibular segments containing the treated teeth were harvested and dissected to remove excess soft and hard tissues. The specimens were fixed in 10% buffered formalin for 72 h. Decalcification was subsequently performed using a formic acid–sodium citrate solution for six months until adequate tissue softening was achieved. After decalcification, the specimens were dehydrated in ascending concentrations of ethanol (70–100%), cleared in xylene, and embedded in paraffin blocks. Individual teeth with their surrounding alveolar bone were separated to obtain discrete tissue specimens. Serial longitudinal sections (4–6 µm thickness) were prepared and stained with hematoxylin and eosin (H&E) and Masson’s trichrome for histological evaluation [30]. Histological sections were examined using a light microscope (Olympus BX51, Olympus Corp., Tokyo, Japan) at ×200 magnification, and representative images were captured using a digital camera system attached to the microscope.

For histomorphometric analysis, the percentage of collagen-positive area was quantified using ImageJ software (version 1.54j, National Institutes of Health, Bethesda, MD, USA). Five non-overlapping microscopic fields were analyzed for each specimen. All histological and histomorphometric assessments were performed by an experienced examiner blinded to the experimental groups to minimize observational bias. The calculated collagen-positive area percentage was used as a descriptive indicator of structural tissue organization and was not intended to represent functional pulp regeneration.

### 2.7. Statistical Analysis

All collected data were organized, tabulated, and statistically analyzed using SPSS software for Windows (version 26.0; IBM Corp., Armonk, NY, USA). The Shapiro–Wilk test was applied to assess the normality of data distribution. Descriptive statistics were expressed as mean ± standard deviation (SD). Paired sample *t*-tests were used to compare measurements at different time points within the same group. Comparisons among multiple groups were performed using one-way analysis of variance (ANOVA), followed by Tukey’s post hoc test for pairwise comparisons. A *p*-value ≤ 0.05 was considered statistically significant.

## 3. Results

### 3.1. Hematoxylin and Eosin

**Figure 1:**
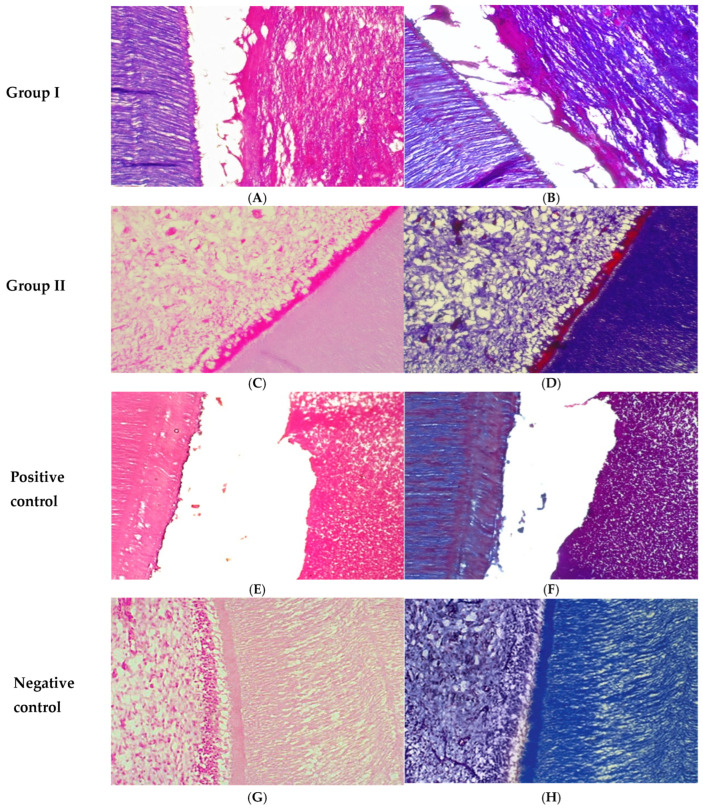
Photomicrographs of the root canals at one month: Group I revealed vital soft tissue, which detached from the dentinal wall (**A**,**B**). Group II displayed pulp-like tissue characterized by irregularly arranged collagen fibers, increased vascularization, and early predentin formation (**C**,**D**). The positive control group exhibited severe inflammatory infiltration (**E**,**F**). The negative control group showed normal architecture of pulp tissue (**G**,**H**). (**A**,**C**,**E**,**G**) Hematoxylin–eosin staining (H&E ×200). (**B**,**D**,**F**,**H**) Masson’s trichrome staining (MTC ×200).

At one month, Group I demonstrated the presence of vital soft tissue that was separated from the dentinal walls. However, the tissue lacked an organized histological architecture, with no evidence of newly formed capillaries or odontoblast-like cells (Figure 1A). In contrast, Group II exhibited pulp-like tissue characterized by irregularly distributed collagen fibers and numerous newly formed blood vessels. An irregular predentin layer was also observed (Figure 1C). The positive control group displayed intense infiltration of acute inflammatory cells occupying the root canal space, with no signs of pulp-like tissue formation or odontoblast-like cells (Figure 1E). Meanwhile, the negative control group presented normal pulp histology, including abundant dispersed blood vessels, numerous collagen fibers, fibroblasts, and a well-organized odontoblastic layer. A thick predentin layer was clearly evident (Figure 1G).

**Figure 2:**
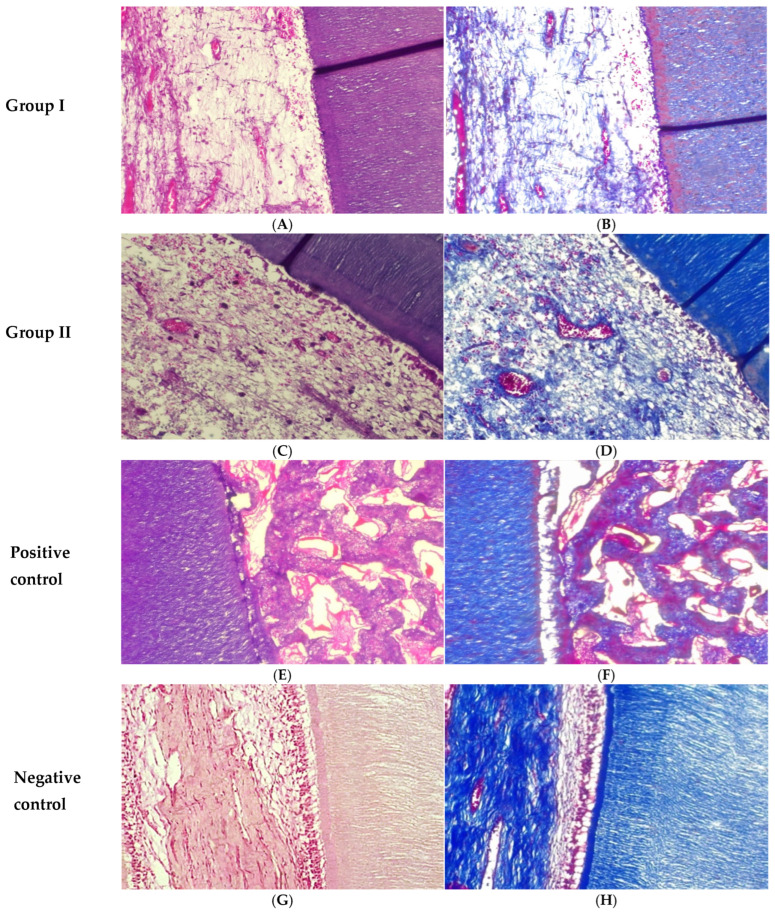
Photomicrographs of the root canals at three months: Group I displayed loose pulp-like tissue (**A**,**B**). Group II revealed more advanced pulp-like tissue with regularly arranged collagen fibers, increased vascularization, and the presence of odontoblast-like cells associated with predentin deposition (**C**,**D**). The positive control group showed bone-like tissue (**E**,**F**). The negative control group exhibited highly organized pulp tissue with dense collagen fibers (**G**,**H**). (**A**,**C**,**E**,**G**) Hematoxylin–eosin staining (H&E ×200). (**B**,**D**,**F**,**H**) Masson’s trichrome staining (MTC ×200).

At three months, Group I showed loosely organized pulp-like tissue containing several blood vessels and a limited number of fibroblasts (Figure 2A). Group II demonstrated more advanced pulp-like tissue formation, with increased vascularization and collagen fibers arranged more regularly compared with group I. Odontoblast-like cells were evident along with a thick predentin layer (Figure 2C). In the positive control group, bone-like tissue formation was observed within the root canal space, while pulp-like tissue and odontoblast-like cells were absent (Figure 2E). The negative control group exhibited well-organized pulp tissue composed of dense collagen fibers, numerous fibroblasts, and blood vessels. A well-defined predentin layer and polarized odontoblasts extending their processes into the dentin were also observed (Figure 2G).

### 3.2. Masson’s Trichrome Staining

At one month, Group I demonstrated extracellular matrix formation with sparse and poorly organized collagen fibers (Figure 1B). In Group II, a greater amount of collagen deposition was observed compared with Group I; however, the fibers remained irregularly arranged (Figure 1D). The positive control group showed minimal collagen content with a disorganized distribution (Figure 1F). In contrast, the negative control group exhibited a well-structured and mature collagen network (Figure 1H).

At three months, Group I displayed a slight increase in collagen deposition, although the fibers remained thin and loosely organized (Figure 2B). Group II showed more mature collagen fibers with a more regular arrangement compared with Group I (Figure 2D). In the positive control group, collagen fibers were sparse and irregularly distributed, indicating an unfavorable environment for pulp tissue regeneration (Figure 2F). Meanwhile, the negative control group demonstrated fully organized pulp tissue characterized by dense and mature collagen fibers (Figure 2H).

**Figure 3:**
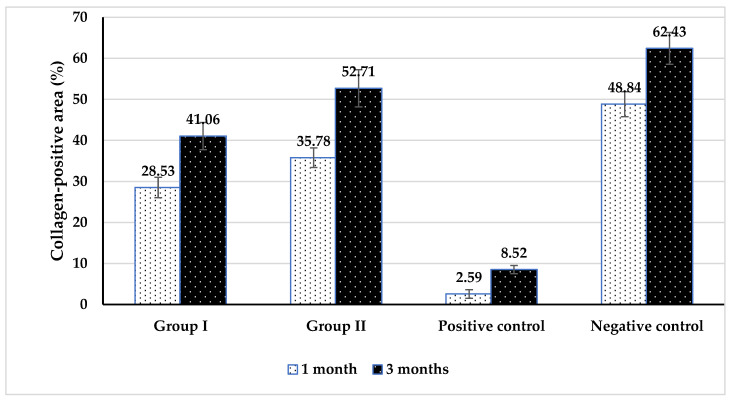
Comparing groups for collagen-positive area (%) at different times.

Histomorphometric analysis, provided in Table 1 and Figure 3, showed a clear intra-group increase over time and strong inter-group differences. Between groups, at both times, the highest mean values were recorded in the negative control, followed by Group II and Group I, while the lowest value was in the positive control. The pairwise comparison showed a significant difference between each group.

Within groups, collagen-positive area percentage increased significantly from 1 month to 3 months for all groups (Group I: 28.53 to 41.06; Group II: 35.78 to 52.71; positive control: 2.59 to 8.52; negative control: 48.84 to 62.43), using paired *t*-tests all *p* < 0.001, indicating significant time effects.

## 4. Discussion

The present study evaluated the effect of 2% chitosan *NPs* used as an irrigating solution during RET of necrotic immature permanent teeth in a dog model. The findings showed that the chitosan NP irrigation protocol resulted in more favorable regenerative outcomes than the conventional NaOCl/EDTA protocol. Histologically, the chitosan group demonstrated earlier and more advanced formation of pulp-like tissue, greater vascularization, better collagen fiber organization, and the appearance of odontoblast-like cells with predentin deposition. Histomorphometrically, this was supported by significantly greater collagen-positive area percentages in Group II than in Group I at both 1 month and 3 months, with progressive improvement over time in all groups. These findings indicate that chitosan *NPs* created a more favorable microenvironment for tissue repair and maturation within the canal space.

A key observation in the present study was that regeneration in the chitosan nanoparticle group was not only greater in quantity, but also better in quality. At 1 month, Group II already exhibited pulp-like tissue with newly formed blood vessels, collagen fibers, and predentin deposition, whereas Group I showed vital soft tissue that remained detached from the dentinal wall and lacked organized architecture or odontoblast-like cells. By 3 months, both groups showed further tissue development, but Group II demonstrated more mature and better organized pulp-like tissue, with more regular collagen arrangement, greater vascularity, and a thicker predentin layer. This pattern suggests that chitosan *NPs* may contribute to accelerated early healing and also support later tissue maturation, rather than merely increasing tissue ingrowth.

The histomorphometric findings reinforce this interpretation. Collagen-positive area increased significantly from 1 to 3 months in all groups, reflecting ongoing extracellular matrix deposition during healing. However, Group II consistently showed higher values than Group I at both time points (35.78% vs. 28.53% at 1 month and 52.71% vs. 41.06% at 3 months), while both remained below the negative control. While collagen deposition alone does not confirm complete pulp regeneration, it serves as an important indicator of extracellular matrix organization and tissue maturation. Therefore, the present findings should be interpreted as indicative of enhanced reparative tissue formation rather than complete functional pulp regeneration. The higher collagen content in the chitosan group, when interpreted together with the histologic findings, supports the conclusion that chitosan NPs promoted more advanced connective tissue remodeling and a more organized regenerative response [31,32,33,34].

The superior performance of chitosan *NPs* may be explained by their combined antimicrobial and bioactive properties. In regenerative endodontics, successful outcomes depend on achieving sufficient canal disinfection without compromising stem cell survival or destroying the dentin-derived signals needed for repair [13,14,15]. Chitosan is known for its biocompatibility, antimicrobial activity, anti-inflammatory effects, and ability to support cell adhesion and extracellular matrix formation [32,33]. In addition, its chelating action may help preserve dentin collagen architecture while maintaining a biologically favorable substrate for cell attachment and signaling. This is particularly relevant in RET, where dentin matrix-associated growth factors contribute to stem cell recruitment, proliferation, and differentiation. These findings are consistent with previous studies demonstrating that chitosan-based irrigation systems promote stem cell viability and proliferation while maintaining a favorable biological microenvironment for regenerative endodontic procedures. Furthermore, this aligns with broader strategies in bioactive material design, where both material composition and structural organization synergistically enhance antimicrobial activity, cell–material interactions, and tissue integration [35,36,37,38].

The nanoscale formulation likely enhanced these effects. Chitosan *NPs* have a high surface-area-to-volume ratio, which may improve their interaction with biofilms and facilitate deeper penetration into dentinal tubules. This may help reduce residual microbial burden while also limiting the persistent inflammatory stimulus that can interfere with tissue organization. The greater vascularization and more regular collagen arrangement observed in Group II may therefore reflect a canal environment that was both better disinfected and more biologically supportive of stem cell-mediated repair. The appearance of odontoblast-like cells and predentin in this group further suggests that chitosan *NPs* may favor not only fibrous tissue ingrowth but also more specialized DPC-like healing [15,16,17,18].

The contrasting findings in the control groups strengthen this interpretation. In the positive control group, severe inflammatory cell infiltration at 1 month and bone-like tissue formation at 3 months indicated that persistent infection redirected healing toward a pathologic or ectopic repair response rather than true pulp-like regeneration. In contrast, the negative control group exhibited the expected normal pulp architecture and the highest collagen values at both time points. Together, these controls provide a useful biological range against which the experimental outcomes can be interpreted: Group II approached a more favorable regenerative pattern, whereas Group I showed partial healing, and the positive control showed failed or aberrant repair.

The blood clot scaffold likely contributed to regeneration in both experimental groups by providing a natural matrix rich in cells and signaling molecules. However, because the scaffolding protocol was similar in both groups, the differences observed are more likely attributable to the irrigation regimen than to the scaffold itself. This is an important point, because it suggests that the irrigant can substantially influence the quality of the regenerative microenvironment even when other procedural steps are standardized [28].

Various techniques have been used to synthesize chitosan *NPs*, including ionotropic gelation, emulsification, microemulsion, polyelectrolyte complexation, solvent diffusion, and reverse micellar methods. In the present study, chitosan *NPs* were prepared using ionotropic gelation, a widely used technique that avoids harsh organic solvents and toxic chemical cross-linkers. This method relies on electrostatic interactions between the positively charged amine groups of chitosan and negatively charged tripolyphosphate, producing biologically compatible *NPs* [21,22]. The 2% concentration was selected because it has been reported to be non-toxic to periodontal ligament stem cells and may facilitate the release of endogenous dentin matrix components, including growth factors involved in stem cell differentiation and proliferation. The present study evaluated a single concentration to standardize experimental conditions, while acknowledging that concentration-dependent effects may influence the physicochemical and biological behavior of chitosan. These characteristics may explain the enhanced regenerative response observed in the present study [18].

Young dogs aged 6–8 months were selected as an experimental model because their immature premolars closely resemble human immature permanent teeth in both anatomical structure and biological behavior. Specifically, canine teeth exhibit similar patterns of apical healing, root development, and inflammatory response to periapical infection, making them highly relevant for studying regenerative endodontic procedures. In addition, the faster growth rate in dogs allows these processes to occur over a shorter timeframe, enabling efficient evaluation of treatment outcomes. From a practical standpoint, the relatively large root canals of dog premolars facilitate endodontic procedures and ensure adequate access for standardized experimental manipulation. Furthermore, the presence of multiple teeth within the same animal permits the collection of sufficient samples while minimizing the number of animals required for the study [27,28,29,30].

Preclinical studies using canine models have historically played an important role in the development and validation of regenerative endodontic procedures and biomaterials prior to clinical application in humans. Several regenerative strategies, including revascularization protocols and bioactive materials such as MTA and scaffold-based approaches, were initially evaluated in animal models and subsequently translated into clinical practice with favorable outcomes. These findings support the relevance of the canine model as a reliable platform for assessing the biological performance and clinical potential of novel regenerative materials.

Instrumentation was intentionally avoided to prevent excessive dentin removal in thin-walled immature teeth, which may increase the risk of fracture, generate a smear layer that occludes dentinal tubules, and potentially compromise the survival of residual stem cells. Consequently, canal disinfection relied primarily on the biological effects of the irrigating solutions. Under these conditions, the superior outcomes observed in the chitosan nanoparticle group suggest that this irrigant may better balance effective disinfection with preservation of the regenerative microenvironment compared with the conventional protocol [27,29].

Despite these promising findings, several limitations should be considered. First, this study was conducted in an animal model, and although dog teeth share similarities with human teeth in dental anatomy and regenerative capacity, differences in metabolism, immune response, and tissue remodeling may limit direct clinical extrapolation. Second, the evaluation periods were limited to 1 and 3 months, representing early and intermediate healing stages rather than long-term functional regeneration. Third, while the collagen-positive area provides valuable information about extracellular matrix deposition and tissue organization, it does not alone confirm complete regeneration of a functional DPC. Therefore, long-term studies incorporating vascular, neural, and functional assessments are needed to further validate these findings. In addition, although chitosan nanoparticles were prepared using dilute acetic acid, the preparation method and subsequent irrigation protocol are expected to minimize residual effects; however, further in vivo and clinical studies are needed to confirm the safety of this formulation in human applications.

From a clinical perspective, the present findings suggest that chitosan *NPs* may represent a promising bioactive irrigant for regenerative endodontic procedures. Compared with the conventional NaOCl/EDTA protocol, chitosan *NPs* were associated with better tissue organization, greater vascularization, and significantly greater collagen deposition, indicating a more favorable environment for healing. Future studies should investigate long-term outcomes, optimize concentration and delivery protocols, and evaluate the translational potential of chitosan *NPs* in human RET. These findings position chitosan *NPs* as a promising candidate for biologically optimized irrigation strategies in regenerative endodontics. The improved performance of chitosan nanoparticles compared with conventional irrigants may be attributed to their combined antimicrobial activity, enhanced penetration, biocompatibility, and ability to support extracellular matrix organization, thereby creating a more favorable microenvironment for regenerative endodontic procedures.

## 5. Conclusions

Within the limitations of this in vivo study, the use of 2% chitosan *NPs* as an irrigating solution during pulp revascularization promoted significantly improved collagen deposition and more organized pulp-like tissue formation compared with the conventional NaOCl/EDTA irrigation protocol. Histological and histomorphometric findings demonstrated enhanced vascularization and extracellular matrix organization in the chitosan nanoparticle group, indicating a more favorable environment for regenerative processes. Although complete regeneration comparable to the normal pulp structure observed in the negative control group was not achieved, chitosan *NPs* exhibited superior biological performance relative to the conventional protocol. These findings suggest that chitosan *NPs* represent a promising bioactive irrigant for RET. Further studies with longer follow-up periods are required to evaluate the physicochemical stability, shelf life, and behavior of chitosan nanoparticles within the biological environment, as well as to validate their safety and efficacy in higher animal models and clinical human applications.

## Figures and Tables

**Table 1:** Comparison of collagen-positive area (%) among the study groups at different evaluation periods.

Group	1 Month	3 Months	Paired *t*-Test	*p*-Value
Group I	28.53 ± 2.48 ^c^	41.06 ± 3.31 ^c^	27.81	<0.01 **
Group II	35.78 ± 2.37 ^b^	52.71 ± 4.53 ^b^	15.00	<0.01 **
Positive control	2.59 ± 1.05 ^d^	8.52 ± 1.02 ^d^	15.93	<0.01 **
Negative control	48.84 ± 3.04 ^a^	62.43 ± 3.89 ^a^	18.22	<0.01 **
ANOVA	341.180	230.524		
*p*-Value	<0.01 **	<0.01 **		

Different superscript letters within the same column indicate statistically significant differences (*p* < 0.05).

## Data Availability

The data presented in this study are available on reasonable request from the corresponding author. The data are not publicly available due to ethical restrictions related to the use of animal experimental data.
